# An Interesting Case and Literature Review of a Coronary Stent Fracture in a Current Generation Platinum Chromium Everolimus-Eluting Stent

**DOI:** 10.1155/2018/4579184

**Published:** 2018-06-04

**Authors:** Sammy San Myint Aung, Htun Latt, Kyaw Kyaw, Chanwit Roongsritong

**Affiliations:** ^1^Institute for Heart and Vascular Health, Renown Regional Medical Center, 1500 E 2nd St No. 302, Reno, NV 89502, USA; ^2^Department of Internal Medicine, School of Medicine, University of Nevada-Reno, 1155 Mill St No. W11, Reno, NV 89502, USA

## Abstract

Coronary interventions are the mainstay of treatment for stenotic coronary vascular lesions. New stent designs are constantly being evaluated to improve stent performances and clinical outcomes. Coronary stent fracture is uncommon; however, it is associated with potential major consequences including acute coronary syndrome and the need for repeated target vessel revascularization due to in-stent restenosis or stent thrombosis. We report a case of a 66-year-old man with an extensive cardiac disease history, who presented with intractable angina and was found to have a fracture of a current generation, platinum chromium everolimus-eluting stent (Synergy, Boston Scientific Inc.).

## 1. Introduction

Coronary stent fracture is defined as a discontinuation of any part of the stent structure seen on angiogram. Stent fracture (SF) was first described with bare-metal stents in 2002 [1]. In 2004, it was subsequently reported with drug-eluting stents [2]. Newer designs, materials, and eluting drugs are being developed to improve the performance, safety, and long-term patency of drug-eluting stents (DES). Synergy stent (Boston Scientific Inc.) is one of the commonly used, current generation of platinum chromium everolimus-eluting stents (EES). To the best of our knowledge, there has been no previously reported case of a stent fracture involving this particular stent.

## 2. Case Presentation

The following case is of a 66-year-old man with a complicated cardiac history, who for some time has been experiencing limiting angina despite being on maximally titrated medical therapy of aspirin, prasugrel, carvedilol, ranolazine, isosorbidemononitrate (sustained release), atorvastatin, lisinopril, nitroglycerin patch, and as needed sublingual nitroglycerin. His cardiac history includes extensive coronary artery disease (CAD).

- Status post coronary artery bypass graft 15 years ago (CABG: reverse saphenous vein graft to first and second obtuse marginal arteries in a sequential fashion and reverse saphenous vein graft to posterior descending artery and posterolateral artery in a sequential fashion)
- Status post redo CABG 2 years ago (CABG: left internal mammary artery [LIMA] to the left anterior descending artery [LAD])
- Status post multiple stents

He also has a history of hypertension, diabetes (on metformin), dyslipidemia, and prior bilateral carotid endarterectomy. He was evaluated by cardiothoracic surgery 8 months prior, and they recommended against reoperation.

**Figure 1:**
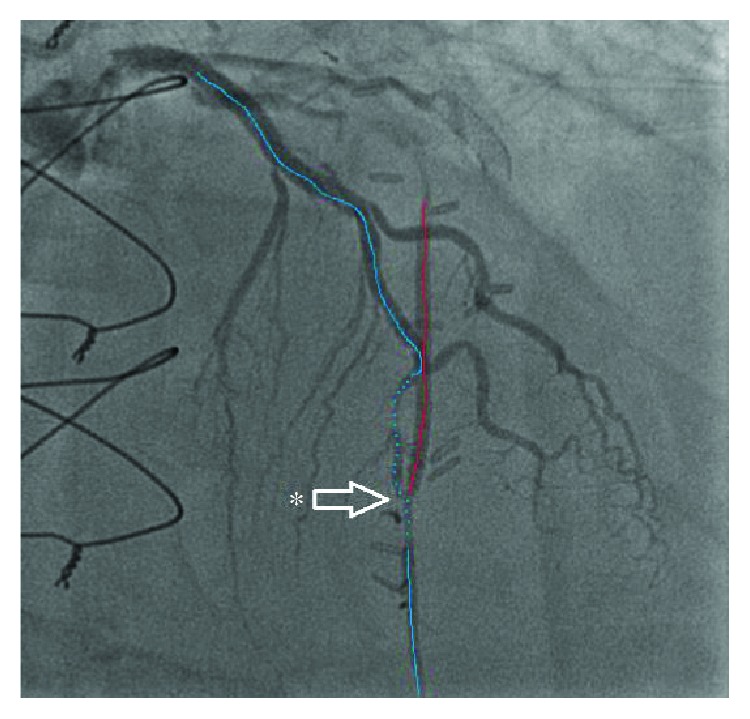
Coronary angiogram showing a long mid-LAD stenosis of 95%, extending from the mid-LAD (dotted blue line) to an area just below the anastomosis with LIMA (white asterisk), with retrograde filling into LIMA (red line).

**Figure 2:**
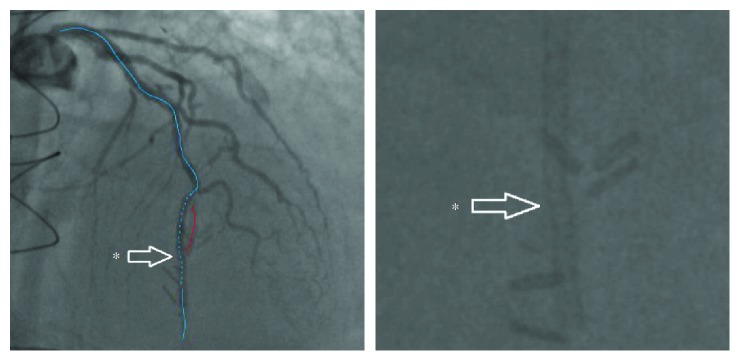
Coronary angiogram showing TIMI III flow over mid-LAD (dotted blue line) and the point of anastomosis (asterisk) after the deployment of Synergy PtCr EES. Note that there is a mild deformity of the stent, which conforms to the coronary anatomy and anastomotic site.

**Figure 3:**
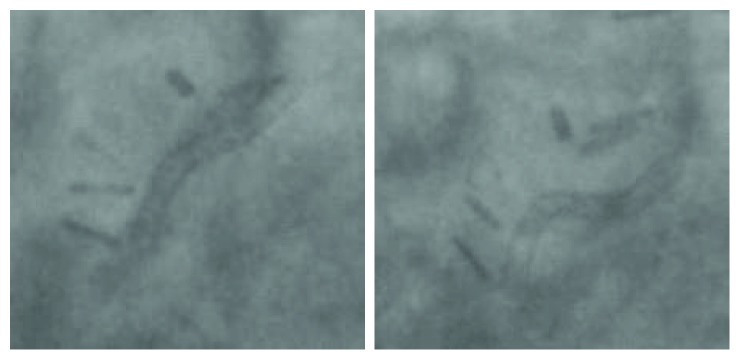
Coronary angiogram showing conformational changes of intact stent during the cardiac cycle (diastole on the left, systole on the right). It can be inferred that various forces are creating a hinge-type motion on the intact stent.

About 4 months prior, he underwent another coronary angiography. This angiogram showed a relatively long 95% stenosis in the mid-LAD. The stenosis extended from the mid-LAD to a bit passed the LIMA anastomosis, with retrograde flow into the LIMA (Figure 1). Some tenting of the LAD at the anastomotic site was also noted. Several predilations were performed at 8 and 10 atmospheres with no significant improvement to the stenosis. A 2.25 × 28 mm Synergy drug-eluting stent (Boston Scientific) was then deployed at 12 atmospheres and postdilated with 2.25 × 20 mm noncompliant balloon at 14 atmospheres. The stent was successfully deployed in the mid-LAD with no residual stenosis. A slight kink in the distal one-third of the stent at the LIMA insertion point was noted (Figure 2).  Figure 3 further demonstrates the movement of the intact stent during diastole and systole. The patient's symptoms temporarily improved.

**Figure 4:**
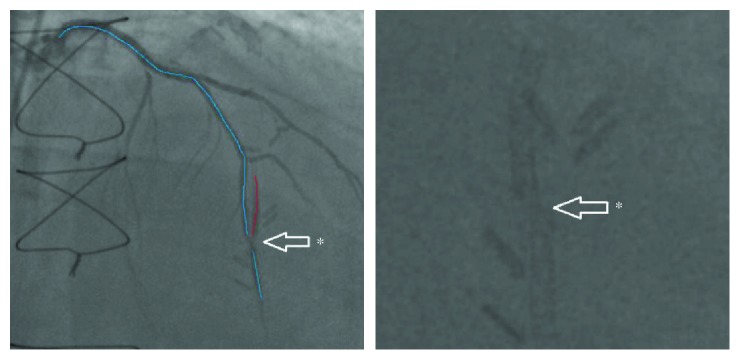
Repeat coronary angiogram showing mid-LAD stent fracture with 70% in-stent restenosis at the site of anastomosis between LAD and LIMA (asterisk).

**Figure 5:**
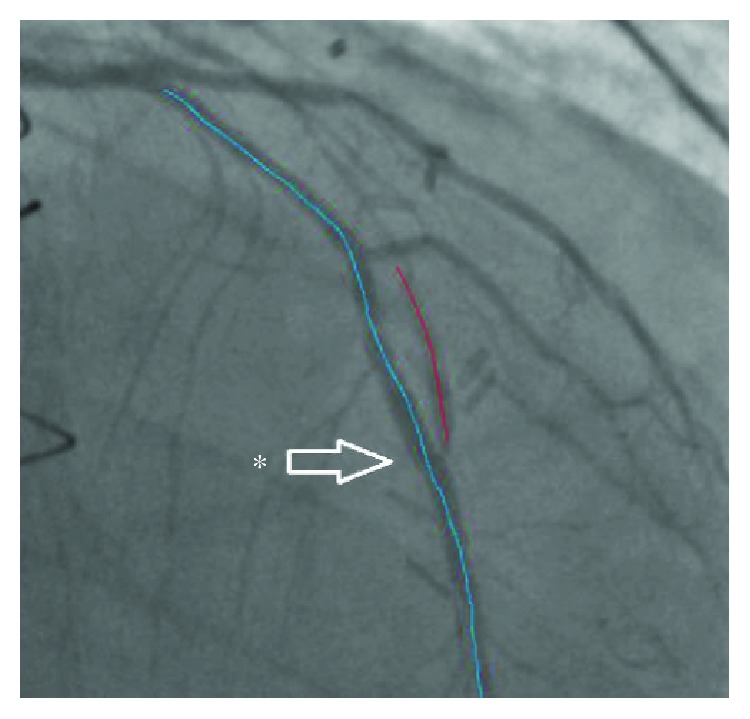
Repeat coronary angiogram showing TIMI III flow in mid-LAD at the point of anastomosis with LIMA (asterisk) after Xience Alpine DES was deployed inside the fractured Synergy DES.

A short time after, he presented again with rest pain. His vital signs were stable. Physical examinations, including cardiac exams, were unimpressive. Electrocardiogram (EKG) showed normal sinus rhythm without significant ST/T changes. Two troponin levels drawn 12-hour apart were both normal. Repeat coronary angiography was performed. It revealed a mid-LAD stent fracture with 70% in-stent restenosis (fractional flow reserve of 0.78) (Figure 4). Coronary intervention was performed and a Xience Alpine DES was deployed inside the fractured Synergy DES with TIMI III flow and no residual stenosis (Figure 5). The patient tolerated the procedure and was discharged in stable condition.

## 3. Discussion

A coronary stent fracture is a discontinuation of any part of the stent structure seen on angiogram [3]. Depending on the study and type of stent, the incidences of SF lie between 0.5% and 19% [4–7]. In a pathological study, the incidence rose as high as 29% [8]. SF rates in sirolimus-eluting stent (SES) were mainly used in these studies. One study by Kuramitsu et al. had shown the incidence of SF in everolimus-eluting stent (EES) to be 2.9% [3]. However, with regard to SF rates, no study has included the Synergy PtCr EES, as yet. Synergy is an EES with a platinum chromium (PtCr) platform. It is a modification from the Promus Element PtCr platform and has thinner struts with a bioabsorbable polymer on the abluminal side [9]. In a randomized trial comparing safety and efficacy, Synergy was shown to be as effective, and slightly better trending, as the Promus element in their clinical endpoints [10].

Coronary SF is thought to be due to a combination of unfortunate factors. These factors are all related to abnormal forces placed on the stent, creating metal fatigue and eventual fracture. Vessel tortuosity (especially in the right coronary artery), vessel calcification, ostial stent location, hinge motion, overlapping stents, increased stent length, smaller stent diameter, coronary aneurysm, and balloon overdilation are some of the known risk factors for SF [3, 4, 6, 7, 11]. The design of the Synergy due to its thin-strut and platinum chromium platform is said to incur flexibility and strength to prevent SF [3, 9]. However, a mechanism for SF in thin-strut DES is longitudinal stent deformation (LSD) [3]. LSD can be caused by balloon overexpansion and abnormalities in the vessel wall such as calcifications [6].

Our patient had experienced intractable chest pain believed to be caused by the in-stent restenosis precipitated by the stent fracture. Clinically concerning complications of coronary stent fractures are acute coronary syndromes (ACS), in-stent restenosis (ISR), stent thrombosis (ST), and the possible need for target vessel revascularization (TVR) [3, 4, 11–13]. These manifestations are related to the disruption of the stent structure and the malapposition of the stent struts to the intimal surface of the vessel. Normally, a DES will provide sufficient amounts of antiproliferative drugs in the vicinity of smooth muscle cells on the vessel intima. However, during a SF, the struts that usually elute the drug are no longer adequately pressed against the vessel walls, thus allowing smooth muscle cells to proliferate. The fractured strut can also mechanically irritate the vessel wall and stimulate smooth muscle cell division, as well as become foci for thrombogenesis [3, 6, 7, 14, 15]. This can lead to a series of cascading events that ultimately disrupts the surrounding hemodynamic environment and lead to the abovementioned complications.

In our case, the SF occurred at the mid-LAD, in the vicinity of the LIMA anastomosis (Figure 4). We believe that the Synergy EES came under a lot of external mechanical strain due to the vessel curvature, as well as the additional tenting force generated by the anastomosis with the LIMA. With each cardiac cycle, the hinge motion (Figure 3), created by the vessel's tortuosity, lead to the eventual metal fatigue and fracture.

The reported incidences of coronary SF may actually be underestimated. Considering the higher incidence reported by Nakazawa et al.'s pathological analysis and the varying incidences of 0.5% to 19% in other studies [4, 6–8], we can infer that a large number of SF are going undetected and are possibly asymptomatic. Like our case, most studies identify SF through angiography [4, 7]. The disadvantage, however, is the lower spatial resolution of angiography compared with other imaging techniques (intravascular ultrasound and optical coherence tomography). Recent technological advancements have introduced the use of stent boost with angiography, a high resolution cine-angiography, that allows easier SF identification [7]. Optical frequency domain imaging has also demonstrated a good ability to detect coronary SFs, in addition to other vessel wall details [16]. Once a SF is identified, classification of the lesion becomes important, for both communication and future management. Lee et al. had used the following classification in their study [15].

- Type I: single strut fracture
- Type II: multiple strut fractures
- Type III: complete transverse fracture with no displacement
- Type IV: complete transverse fracture with displacement

Based on this, our case would be considered a type IV fracture, the most severe form. There is no formal management guideline for treating coronary SF. In a single-center study by Lee et al., they proposed to treat SF with no signs of ISR with dual or triple antiplatelet therapy for an unknown duration of time. Significant ISR with severe SF (type II to type IV) warranted coronary intervention [15]. DES use in the treatment of SF was shown to have lower rates of target vessel revascularization than plain old balloon angioplasty [17]. Our patient was managed in a way consistent with the recommendations above. We treated our patient with a repeated coronary intervention using the Xience Alpine EES. Although overlapping stents are known to be a risk factor for further SF, it was important that such a severe stent fracture and in-stent restenosis be amended in an attempt to return maximal perfusion to distal tissues.

## 4. Conclusion

Regardless of stent type and design, stent fractures are a complication that should always be considered. Vessel anatomy, hinge motions, overlapping stents, calcifications, and stent types are a few of the risk factors mentioned that should be incorporated in the decision process prior to performing coronary intervention. Having a simple yet accurate description of stent fractures based on image modality will help in standardizing appropriate treatment guidelines of coronary SF. Further studies on the clinical outcomes in patients who received stent-within-a-stent treatment, as our patient, would help in determining if this is the best treatment option for coronary SF.
